# Do NAAT-Based Methods Increase the Diagnostic Sensitivity of *Streptococcus agalactiae* Carriage Detection in Pregnant Women?

**DOI:** 10.3390/diagnostics13050863

**Published:** 2023-02-23

**Authors:** Agnieszka Sroka-Oleksiak, Wojciech Pabian, Joanna Sobońska, Kamil Drożdż, Tomasz Bogiel, Monika Brzychczy-Włoch

**Affiliations:** 1 Department of Molecular Medical Microbiology, Chair of Microbiology, Faculty of Medicine, Jagiellonian University Medical College, 31-121 Krakow, Poland; 2 Clinical Department of Gynecological Endocrinology and Gynecology, Jagiellonian University Medical College, 31-501 Krakow, Poland; 3 Department of Microbiology, Ludwik Rydygier Collegium Medicum in Bydgoszcz, Nicolaus Copernicus University in Toruń, 85-094 Bydgoszcz, Poland; 4 Department of Clinical Microbiology, Antoni Jurasz University Hospital No. 1, 85-094 Bydgoszcz, Poland

**Keywords:** GBS, PCR, pregnant women, primers, real-time PCR, *Streptococcus agalactiae*

## Abstract

The aim of the study was to evaluate particular polymerase chain reaction primers targeting selected representative genes and the influence of a preincubation step in a selective broth on the sensitivity of group B *Streptococcus* (GBS) detection by nucleic acid amplification techniques (NAAT). Research samples were vaginal and rectal swabs collected in duplicate from 97 pregnant women. They were used for enrichment broth culture-based diagnostics, bacterial DNA isolation, and amplification, using primers based on species-specific *16S rRNA*, *atr* and *cfb* genes. To assess the sensitivity of GBS detection, additional isolation of samples preincubated in Todd-Hewitt broth with colistin and nalidixic acid was performed and then subjected to amplification again. The introduction of the preincubation step increased the sensitivity of GBS detection by about 33–63%. Moreover, NAAT made it possible to identify GBS DNA in an additional six samples that were negative in culture. The highest number of true positive results compared to the culture was obtained with the *atr* gene primers, as compared to *cfb* and *16S rRNA* primers. Isolation of bacterial DNA after preincubation in enrichment broth significantly increases the sensitivity of NAAT-based methods applied for the detection of GBS from vaginal and rectal swabs. In the case of the *cfb* gene, the use of an additional gene to ensure the appropriate results should be considered.

## 1. Introduction

The recto-vaginal colonization of *Streptococcus agalactiae* in pregnancy is estimated at approximately 10–40% and constitutes one of the greatest risk factors of premature birth or the development of sepsis, meningitis and pneumonia among newborns [1,2,3,4,5]. Since 1996, the American College of Obstetricians and Gynecologists (ACOG) followed by the Centers for Disease Control and Prevention (CDC) and the American Academy of Pediatrics (AAP) has recommended screening for group B *Streptococcus* (GBS) carriage in all pregnant women between 35 and 37 weeks of gestation and intrapartum antibiotic prophylaxis in case of GBS-positive results [1]. 

In microbiological diagnostics, the gold standard for GBS screening is incubating specimens in enrichment broth and then culturing them on solid media. Although these methods are simple and inexpensive, they are limited by their time-consuming nature and lower sensitivity compared to molecular methods. The American Society for Microbiology (ASM) recommended the additional application of nucleic acid amplification techniques (NAAT)-based methods, usually polymerase chain reaction (PCR) variants [1]. On the other hand, the CDC approved real-time PCR assays targeting the *cfb* gene (which encodes CAMP factor) directly from samples if the results of culture are negative or not available [6]. Moreover, the research by Tickler et al. describes cases of obtaining negative results for GBS colonization in PCR based on the detection of the *cfb* gene accompanying positive results in the culture. This is related to the presence of GBS strains with a chromosomal deletion in the region of the *cfb* gene [7]. There are also other genes are also used in GBS detection by molecular methods, e.g., *atr*, *sip*, *sod*A, *scp*B or *16S rRNA* [8]. Each of them, depending on the tests performed, is characterized by a different degree of sensitivity and specificity. Based on the available literature data, it was found that of the genes listed above, except for *cfb*, the *atr* gene is the most commonly used [9,10,11,12,13,14,15].

False-negative results from culture or non-standardized NAAT methods can result in delayed treatment, increasing the risk of serious neonatal and maternal infections and/or mortality [1,10]. Hence, it is crucial to develop existing or novel diagnostic methods to prevent complications and improve outcomes. 

The aim of this study was to evaluate the sensitivity of GBS detection in a group of pregnant women by NAAT-based methods on *cfb* and *atr* genes and the *16S rRNA*-conserved gene in comparison to the enrichment broth culture. The second goal was to assess the sensitivity of NAAT-based methods with and without preincubation in selective Todd-Hewitt (TH) broth supplemented with colistin and nalidixic acid.

## 2. Materials and Methods

### 2.1. Patients

The study included 97 pregnant women aged 23–40 years old from whom vaginal and rectal swabs were collected in different trimesters of pregnancy. The inclusion criteria used for the recruitment of patients to the investigation were the following: age between 18 and 40 years old, pregnancy, lack of antibiotic or probiotic use for up to 30 days before getting pregnant and during pregnancy and written consent to participate in the research.

The pregnant women who met at least one of the following conditions were excluded from the study: autoimmune diseases, immune disorders, a high-risk pregnancy, rupture of the membranes, premature birth or clinical symptoms of urinary tract infection.

The study has been approved by the Bioethics Committee (No. KBET/1072.6120. 51.2017) and conducted in accordance with the provisions of the Declaration of Helsinki.

### 2.2. Samples

**Figure 1:**
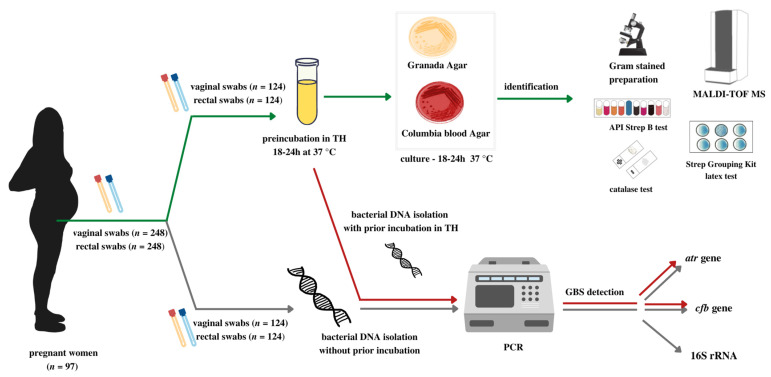
The scheme shows the course of the research and the methods used for GBS–carriage diagnostics in the studied samples. Green and red colors are, respectively, the classical and molecular course of diagnostics in accordance with ASM recommendations [1], taking into account preincubation in liquid TH medium with colistin and nalidixic acid. Gray color is the course of GBS identification using molecular methods, without the preincubation step.

The research material consisted of swabs from the lower vagina (vaginal introitus) (*n* = 124) and rectum (*n* = 124) collected in duplicate from all recruited pregnant participants (*n* = 97) during a routine prenatal medical visit. The first vaginal and rectal swabs were placed in a non-nutrient Amies transport medium (Eurotubo) and the second swabs in 2 mL of 0.9% NaCl. The research samples were delivered to the Department of Microbiology, Jagiellonian University Medical College. Each time, the first vaginal and rectal swabs were used for the culture diagnostics according to ASM recommendations [1], while the second swabs were used for bacterial DNA isolation (Figure 1).

### 2.3. The Culture and Other Identification Tests

The swabs were preincubated in the TH liquid medium supplemented with colistin and nalidixic acid (Becton Dickinson, Microbiology Systems, Cockeysville, MD, USA) for 18–24 h at 37 °C in aerobic conditions and then transferred to a solid medium, including Columbia blood agar with 4% sheep blood (Becton Dickinson, Microbiology Systems, Cockeysville, MD, USA) and Granada medium (Becton Dickinson, Microbiology Systems, Cockeysville, MD, USA). The cultures were incubated for 18–24 h at 37 °C under aerobic conditions. The growing colonies were subjected to classical species identification towards GBS (assessment of Gram-stained preparations, presence of catalase with 3% hydrogen peroxide), Streptococcal Grouping Kit latex test (Becton Dickinson, Microbiology Systems, Cockeysville, MD, USA), and finally API Strep test (bioMérieux, Marcy-l’Étoile, France) and/or MALDI-TOF-based identification.

After microbiological identification, the swabs in the TH medium (after preincubation) were frozen at −80 °C in order to preserve the materials for possible further tests.

### 2.4. Isolation of Bacterial DNA

Bacterial DNA was extracted using the manufacturer’s protocol for the EURx Bacterial and Yeast Genomic DNA Purification Kit (EURx, Gdańsk, Poland). Additionally, during the enzymatic lysis step, 7 µL of lysozyme (50 mg/µL, Sigma-Aldrich, St. Louis, MO, USA) and 3.5 µL of mutanolysin (2 U/µL, Sigma-Aldrich, St. Louis, MO, USA) were added to the samples.

To assess the sensitivity of GBS detection, an additional isolation of bacterial DNA was performed and then “de novo” amplified, after samples preincubation in TH broth supplemented with colistin and nalidixic acid (Figure 1).

### 2.5. PCR Protocols

The isolates were used to perform a PCR amplification to verify the presence of *S. agalactiae* DNA in each sample. Based on the literature data, three pairs of primers were selected: the GBS species-specific *16S rRNA* gene—F1-IMOD [16], *atr*—encoding the glutamine transport protein of *S. agalactiae* [17] and *cfb*—encoding the CAMP factor in *S. agalactiae* [18]. The sequences of all primers are shown in Table 1.

The standardization of the reaction conditions was carried out with the use of a DNA sample extracted from the *S. agalactiae* ATCC 12386 reference strain. 

Conventional PCR was used for the detection of GBS species-specific *16S rRNA* and *atr* genes. The total volume of the reaction mixture was 20 µL and consisted of 4 μL of Silver Taq Polymerase (Syngen, Wrocław, Poland), 0.6 μL of forward and reverse primers at the concentration of 10 µM each, 11.8 μL of nuclease-free water (A & A Biotechnology, Gdańsk, Poland) and 3.0 μL of DNA extracts. The amplification was performed in a T-100 thermocycler (BioRad, Hercules, CA, USA) using the following thermal profile (the same for both *16S rRNA* and *atr* genes primers): 95 °C–15 min, 35 cycles (95 °C–20 s, 55 °C–60 s, 72 °C–60 s) and final elongation at 72 °C for 10 min. In all the amplification rounds, positive and negative controls were used (DNA of GBS reference strain and nuclease-free water, respectively). Next, 5 µL of each amplicon was subjected to electrophoretic separation in 1.5% agarose gel (Prona ABO, Gdańsk, Poland) in a 1× concentrated TBE running buffer (Sigma-Aldrich, St. Louis, MO, USA). The amplicons were visualized in the FastGene^®^ FAS-Digi PRO (Nippon Genetics Europe, Düren, Germany) gel documentation system. The size of the product for the *16S rRNA* was 405 bp, while for the *atr* gene it was780 bp; a marker of DNA size ranging from 100 to 1000 bp (A & A Biotechnology, Gdańsk, Poland) was used. 

A real-time PCR method was applied to detect the *cfb* gene. The total volume of the reaction mixture was 25 µL and consisted of 12.5 μL of RT HS-PCR Mix probe (A & A Biotechnology, Gdańsk, Poland), 0.5 μL of each primer (10 µM), 0.5 μL of fluorescent probe (10 µM), 8.5 μL of nuclease-free water (A & A Biotechnology, Gdańsk, Poland) and 2.5 μL of DNA extracts. The amplification was performed in CFX96 thermocycler (BioRad, Hercules, CA, USA) using the following thermal profile: 95 °C–10 min, 40 cycles (95 °C–15 s and 60 °C–60 s). 

The statistical analyses were carried out using STATISTICA version 13.3 with the Medical Kit, version 4.0.67. The McNemar test was used for the evaluation of statistically significant differences between each method, and the Cochran Q test with Dunn’s post-hoc test were used to compare the results obtained from the three methods. The level of significance was set at α = 0.05.

## 3. Results

The study involved 97 pregnant patients, from whom vaginal and rectal swabs were collected in different trimesters of pregnancy. The majority of the samples were collected in the third trimester of pregnancy (*n* = 84), then in the first trimester (*n* = 23) and the second trimester (*n* = 17). Some patients were studied in both the first and third trimesters or in the second and third trimesters, hence it was possible to collect material twice at different stages of pregnancy from the same patient. 

### 3.1. Diagnostics of Patients with the Use of Classic Microbiological Methods

In standard microbiological diagnostics, 27 vaginal and 22 rectal samples were GBS-positive, while the remaining 97 vaginal and 102 rectal samples were negative. 

### 3.2. Diagnostics of Patients with the Use of Standard NAAT-Based Methods

**Figure 2:**
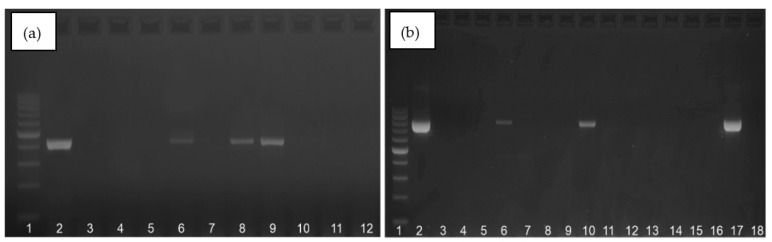
Exemplary results of electrophoresis for GBS detection based on *16S rRNA* gene—405 bp (**a**) and *atr* gene—780 bp (**b**). First paths on (**a**,**b**)—DNA size marker (100–1000 bp, A & A Biotechnology, Gdańsk, Poland), second paths—positive control (DNA isolated from *S. agalactiae* ATCC 12386), other paths—the tested samples. Paths number 12 (**a**) and number 18 (**b**)—negative controls—nuclease-free water (A & A Biotechnology, Gdańsk, Poland).

The amplification products for *16S rRNA* gene and atr gene are presented in the Figure 2a,b. In each of the PCR methods used, a band was obtained at the appropriate height for the positive control—the reference strain S. agalactiae ATCC 12386—and no band for the negative control (DNase and RNase-free water), which proves that the appropriate conditions and course of the amplification reaction were used.

**Figure 3:**
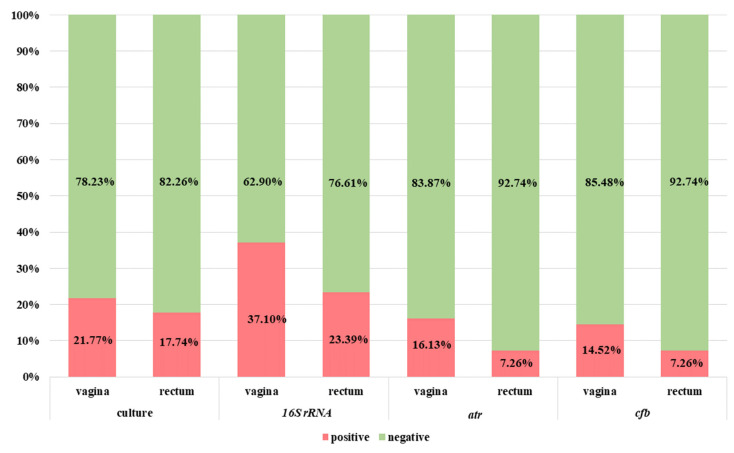
The comparison of GBS identification results using the culture method in TH selective medium and NAAT-based methods (based on the *cfb*, *atr* and *16S rRNA* conserved gene) directly from clinical materials without the preincubation step.

The summary results for GBS detection by PCR (with the use of *16S rRNA*, *atr* and *cfb* genes-targeting primers) performed directly from clinical materials without previous incubation in TH broth with antibiotics are presented in Figure 3.

The positive amplification results for *16S rRNA* conserved genes were observed in the case of 37.10% (*n* = 46) vaginal and 23.39% (*n* = 29) rectal samples (Figure 3). Meanwhile, twenty-seven samples (nineteen vaginal and eight rectal) were true positive (in accordance with the enrichment broth culture method which is a gold standard). False-negative results were related to nine vaginal and thirteen rectal samples. 

**Figure 4:**
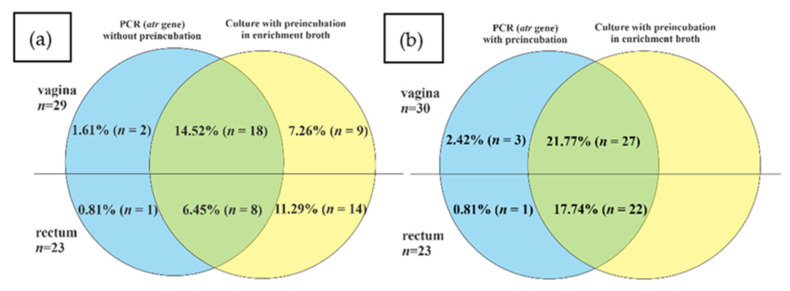
The comparison of GBS identification results by the culture method without preincubation in enrichment broth and by PCR method for *atr* gene (**a**) and with (**b**) preincubation in TH selective broth: blue, percentage/number of positive samples only in PCR; green, percentage/number of positive results both in culture method and PCR (true positive); yellow, percentage of positive results in culture method only.

The positive results for *atr* gene presence were detected in the case of 16.13% (*n* = 20) vaginal and 7.26% (*n* = 9) rectal samples (Figure 3). Among them, respectively, eighteen (90.0%) vaginal and eight (88.89%) rectal samples were true positive. Moreover, twenty-three samples (nine vaginal and fourteen rectal) were positive in culture but negative in PCR (interpreted as false negative). Meanwhile, three additional samples (two vaginal and one rectal) were negative in culture but positive for *atr* gene in the PCR method (Figure 4a). 

**Figure 5:**
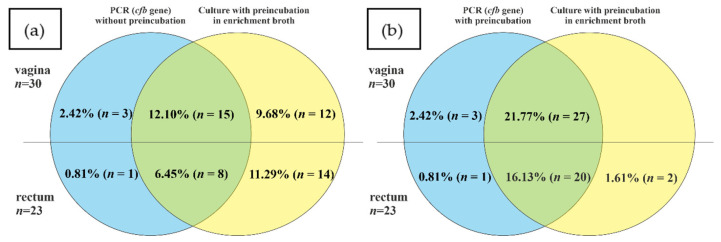
The comparison of GBS positive identification results by the culture method without preincubation in enrichment broth culture method and real-time PCR method for the *cfb* gene (**a**,**b**) preincubation in TH selective broth:. blue, percentage/number of positive samples only in real-time PCR; green, percentage/number of positive results both in culture method and real-time PCR (true positive), yellow, percentage of positive results in culture method only.

The results with th use of *cfb* gene were positive for 14.52% (*n* = 18) vaginal and 7.26% (*n* = 9) rectal samples (Figure 3). Among them, respectively, 83.3% (*n* = 15) vaginal and 88.89% (*n* = 8) rectal samples were true positive. Meanwhile, 20.97% (*n* = 26) of samples were false negative (12 vaginal and 14 rectal). Moreover, four samples were detected as *cfb* positive (of which two were also positive for *atr* gene) but negative in the culture method (Figure 5a).

In order to improve the sensitivity of GBS detection by PCR and real-time PCR with the use of *atr* and *cfb* primers, additional isolation of bacterial DNA from the TH medium after the preincubation step was performed. After this additional step, for *atr* primers, all the samples positive in culture were also detected by PCR. Moreover, one additional vaginal sample was positive in PCR, but negative in culture (Figure 4b). The application of preincubation in an enrichment broth medium also improved the detection of GBS with an application of *cfb*-targeting primers and increased the proportion of positive results by 9.68% (*n* = 12) in vaginal and by 9.68% (*n* = 12) in rectal samples, but still 1.61% (*n* = 2) samples were false negative for the *cfb* gene (Figure 5b).

**Figure 6:**
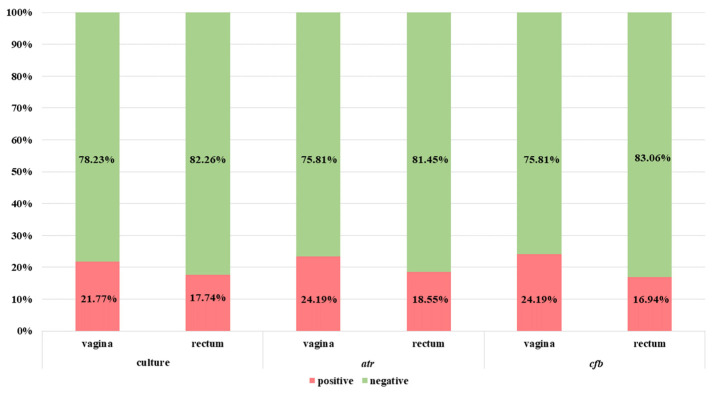
The comparison of GBS identification results using the culture method with preincubation in enrichment broth and NAAT-based methods with preincubation based on the *atr* gene (conventional PCR) and *cfb* gene (real-time PCR).

The summary results for the *cfb* and the *atr* genes detection after preincubation are shown in Figure 6. Due to the large discrepancies in the results with the use of species-specific *16S rRNA* primers in relation to the culture method with preincubation in enrichment broth and primers tested in this study (for *atr* and *cfb* gene), we decided not to make this comparison. The discrepancies were most probably due to a close relatedness (*16S rDNA* sequences) between some other Gram-positive cocci and the inability of the applied primers to distinguish between them and amplify *S. agalactiae* DNA exclusively.

### 3.3. Statistical Analysis

The observed differences in the detection of GBS in samples without preincubation in TH broth with antibiotics for the *atr* and *cfb* genes were not statistically significant (*p* = 1.00) in contrast to the results obtained for *16S rRNA* (*p* < 0.000001) also in relation to the culture method with the preincubation in enrichment broth (*p* < 0.000004).

This observation had a direct impact on the sensitivity and specificity, which were presented in Table 2. The application of the preincubation step before bacterial DNA isolation for PCR purpose allowed for a sensitivity of 100% for the *atr* gene (both in vaginal and rectal samples), compared to 100% (vaginal samples) and 90.90% (rectal samples) for the *cfb* gene (Table 2).

Summarizing, the use of 18–24 h preincubation in a TH broth with antibiotics increases the sensitivity of GBS detection in PCR by about 33–63% (33.30–63.60% for *atr* primers and 44.40–54.50% for *cfb* primers).

### 3.4. Comparison of NAAT and Culture Methods in Relation to GBS Carriers

Additionally, for epidemiological purpose, an analysis of GBS carriers’ status in the third trimester of pregnancy was performed, according to the ASM recommendations [1]. In our study among all 97 pregnant patients, 84 were in the third trimester of pregnancy, which were further analyzed. Based on the NAAT, after preincubation in TH broth, twenty-one (25%) patients were identified as GBS carriers–three more compared to NAAT-based methods directly from clinical materials and two more compared to the gold standard (Table 3). 

## 4. Discussion

In our study, we assessed the sensitivity of selected NAAT-based methods (PCR and real-time PCR) with the use of three different target genes (*16S rRNA*, *atr* and *cfb*) to detect of GBS from vaginal and rectal swabs with preculture in selective broth and compared to direct testing of clinical samples. All the results were compared to the culture method with the preincubation in enrichment broth – the gold standard in microbiological diagnostics [1].

Our research clearly supports the necessity to use the preincubation step (despite the fact of extending the time required to obtain the final results) before performing molecular tests, regardless of the targeted gene (*atr*, *cfb*, etc.). This has a direct impact on the increase of sensitivity by about 33–63%. After the preincubation step (for 18–24 h in TH broth medium), we showed higher sensitivity of *atr*-targeting primers for GBS detection in comparison to the *cfb* and *16S rRNA*-directed primers. The preculture did not have an effect on the specificity of detection by *atr* and *cfb* primers, which was similar to these noted before preincubation.

Based on the literature data concerning the identification of GBS by molecular methods, *cfb* is the most frequently used gene [9,10,11,12,13,14,15]. Although the obtained sensitivity and specificity of GBS detection based on *cfb* gene investigation, as it has been shown previously, is relatively high, our research shows that these values may be lower when compared with the sensitivity of other genes, e.g., *atr*. Similar conclusions can be drawn about the basis of the results in the research by Carrillo-Avila et al. [19] and Mousavi et al. [20]. For example, in the studies carried out by the team of Carrillo-Avila the number of positive results using the *cfb* gene was 73, and for the *sip* gene it was 75, when performed on 78 samples which indicated positive by culture. This resulted in sensitivity and specificity at the levels of 93.58% and 94.62% for *cfb* and 96.15% and 95.45% for *sip* gene, respectively [19]. In the previous study, the sensitivity and specificity for the *cfb* gene-based investigation were estimated at the level of 73.33% and 87.23%, while for the *atr* gene—80% and 86.70%, respectively [20]. 

Although the obtained differences in the results between genes are not statistically significant, in the context of individual pregnant patients who receive false-negative results, this is of great importance. At this point, it is also worth emphasizing that some commercial tests or automatic systems use the *cfb* gene-based methodology for GBS detection. For example, in the research by Vieira et al., in addition to the real-time PCR and culture methods, Xpert^®^ GBS rapid test (Cepheid, Sunnyvale, CA, USA) was used [21]. This device enables automatic isolation, purification and amplification of the target sequence for the *cfb* gene by qPCR. The short time from the isolation to the results, which is only 50 min, favors the use of this method. However, the sensitivity and specificity of Xpert GBS in comparison to qPCR were, respectively, 53.2% and 93%. By contrast, for the reference culture method the figures were 61.80% and 75.80%, respectively [21]. In the subsequent studies, the use of Xpert GBS allowed for much higher sensitivity (86.70%) and specificity (95.60%) of the results, but the reference culture method was applied only for this comparison and no additional molecular tests were performed. Nevertheless, it was also noted that Xpert GBS can generate errors caused by factors such as excess mucus or feces causing inhibition of the PCR reaction and influencing the microfluidic channel in the cassette [22]. Furthermore, in the work by Helmig et al. with the use of Xpert GBS, the sensitivity and specificity of detection were estimated at 100% (86.28–100%) and 97.50% (91.26–99.70%) respectively, and the positive predictive value was 92.60%. However, for one patient, no result was obtained despite retesting [23]. An alternative to commercial GBS identification tests may be IDI-Strep B kit (IDI, Sainte-Foy, QC, Canada), which also targets the *cfb* gene. The sensitivity and specificity of this test are comparable to Xpert GBS. However, due to the high cost of testing, these platforms can only be used for the detection of maternal intrapartum colonization [24]. 

Therefore, in the routine diagnostics of GBS carriage, in addition to culture, the use of PCR targeting *cfb* gene and additionally another gene should be considered. The *atr* gene tested in our research is a good candidate, which has also been previously confirmed by the research of other authors. In the work by de-Paris et al. [25], positive results with the use of the culture method were obtained in 42 (15.96%) samples, and the use of particular genes primer sequences enabled the detection of the *atr* gene in as many as 71 (26.99%) of GBS-positive samples. All the samples with positive results in culture were also positive in the PCR method, so the sensitivity of the PCR compared to the gold standard was 100%. Obtaining this high sensitivity was possible thanks to the application of the preincubation step in selective enriched broth before PCR was performed. Nevertheless, in other studies, the specificity for the detection of the *atr* gene by PCR was from 82.60% [26], through 85.60% [27] to 100% [28]. On the other hand, the specificity of the molecular methods compared to culture was estimated at 73.10–73.60% vs 95.60% [28] with a negative predictive value of 100%, which is important in the context of obtaining negative results that are true negatives. This is then equivalent to skipping antimicrobial prophylaxis during a labor [29]. Based on the literature data, no commercial PCR test based on the *atr* gene has been reported so far.

For both the *atr* and *cfb* genes, the total number of GBS positive results in the vaginal samples was 30, while in the rectum samples it was 23 (for the *atr* gene) and 21 (for the *cfb* gene). There were three discrepancies within the samples group—one of them was positive for the *atr* gene and negative for *cfb*, while two other samples were vice versa. Although the differences between the results obtained for the *atr* and *cfb* genes detection were not statistically significant, in this situation it should be justified to consider application of methodology based on the use of a third gene to conclude the results. A negative result for *cfb*, while being positive for another GBS specific gene, could be the reason for the chromosomal deletion of this gene, which was described in the introduction [7]. Therefore, the obtained results would be false negative in relation to culture methods. This would explain our results for two rectal samples, which were positive in the culture and for the *atr* gene but simultaneously negative for the *cfb*, despite the use of preincubation and reamplification steps. 

The use of species-specific *16S rRNA*-encoding gene primers in amplification without preincubation significantly increased the percentage of positive results in relation to those obtained with the use of *atr* and *cfb* genes and culture methods. Undoubtedly, the advantage of primers based on *16S rRNA* is the high probability of GBS detection, but it can result in generating false-positive results, which is also confirmed by our research. The sequences of these primers were chosen from a veterinary publication on the detection of GBS in milk [16], but according to the authors, using a pair of F1-IMOD primers, a large collection of *S. agalactiae* isolates, including bovine and human isolates, as well as reference strains were identified in 100% [16]. The online sequence analysis showed that the primers also enable the detection of other species belonging to *Streptococcus* spp. which explains the significantly higher sensitivity and low specificity in relation to the other molecular methods and the culture method. An additional argument supporting the above explanation is the source of the collected samples—vagina and rectum, which are ecosystems abundant in various species of bacteria, including the *Streptococcus* spp. representatives. Nevertheless, this genus is one of the dominant groups of bacteria also in human milk [30]. 

Based on these examples, one of the limitations of current methods (in addition to those mentionedbefore) is the abundance of amplification methods, manifested in the use of different primer pairs—allowing for amplification of different genes or GBS genome regions, which also translates into different results. 

Comparing the PCR results to the gold standard (culture method according to ASM recommendation), in our study we obtained five positive results for the *cfb* and *atr* genes (two of them were positive for both genes, one for the *atr* gene only and two for the *cfb* gene only), which were negative in culture. This observation can be the result of a low number of bacterial cells (cfu/mL—colony forming unit) or vaginal colonization by GBS at the low level, damaged cells and presence of DNA fragments only. Other explanations could be the presence of other bacteria in the clinical material that constitute the vaginal microbiota, but inhibit the growth of GBS despite the use of selective broth in the culture [25]. For example, the growth of *S. agalactiae* in TH broth with gentamicin and nalidixic acid may be inhibited by the presence of bacteria of the *Enterococcus* genus [31], which may explain the negative results in culture and positive results for these samples in PCR/real-time PCR method. In order to avoid this situation, in our study we used a preincubation step in TH broth with colistin (instead gentamicin) and nalidixic acid. The discrepancies in our results of culture and molecular methods may result from the presence of small numbers of bacterial cells in the tested material or sample collection procedure itself. The culture method might then have been insufficient for GBS detection [22,27]. In addition, there are GBS strains that do not show hemolysis on the Columbia blood agar media, which makes their identification difficult and has a direct impact on discrepancies while NAAT-based methods are applied (compared to culture as a reference) [24]. However, in our study Granada medium was also used to exclude colonies with a different phenotype. Considering the dynamics of genetic variation in *S. agalactiae* strains, which may manifest in different phenotypes, the implementation of molecular methods should be considered as an auxiliary tool in the routine diagnostics of GBS carriage. 

Moreover, the future direction of the current method should be based on the development of a universal multiplex PCR to simultaneously detect GBS species and resistance genes. This is important in light of the increasing resistance to macrolides and clindamycin and the limitations of current molecular methods. NAAT-based assays could play an important role and their results could support rapid and effective therapy.

## 5. Conclusions

This study demonstrated that in the *S. agalactiae* detection by PCR methods, a greater diagnostic value lies in primers enabling the amplification of genes specific for this species: *atr* and *cfb* in relation to primers based on *16S rRNA* genes. Moreover, the use of 18–24 h preincubation in TH broth with appropriate antibiotics significantly increases the sensitivity in the detection of GBS carriers by NAAT-based methods compared to samples derived directly from clinical material.

## Figures and Tables

**Table 1:** Sequences of primer pairs and probe specific for *S. agalactiae* (GBS) used in this study.

Primer Pairs	Sequence 5′ → 3′	Source
*16S rRNA*	F: GAGTTTGATCATGGCTCAG	[16]
	R: ACCAACATGTGTTAATTACTC	
*atr*	F: CGATTCTCTCAGCTTTGTTA	[17]
	R: AAGAAATCTCTTGTGCGGAT	
*cfb*	F: GGGAACAGATTATGAAAAACCG	[18]
	R: AAGGCTTCTACACGACTACCAA	
	P: FAM-AGACTTCATGCGTGCCAACCCTGAGAC-3′-BHQ1	

**Table 2:** The sensitivity and specificity of the results for tested primer pairs (*16S rRNA* and *atr*–conventional PCR and *cfb*–real-time PCR) in accordance with culture as a reference method.

	Gene	Source	Sensitivity	Confidence Interval	Specificity	Confidence Interval
PCR from samples without preincubation in TH enrichment broth	*16S rRNA*	vagina	70.4%	(49.8–86.2%)	72.2%	(62.1–80.8%)
		rectum	40.9%	(20.7–63.6%)	80.4%	(71.4–87.6%)
	*atr*	vagina	66.7%	(46.0–83.5%)	97.9%	(92.7–99.7%)
		rectum	36.4%	(17.2–59.3%)	99%	(94.7–100%)
	*cfb*	vagina	55.6%	(35.3–74.5%)	96.9%	(91.1–99.4%)
		rectum	36.4%	(17.2–59.3%)	99%	94.7–100%)
PCR from samples preincubated in TH enrichment broth	*atr*	vagina	100%	(87.2–100%)	96.9%	(91.2–99.4%)
		rectum	100%	(84.6–100%)	99%	(94.7–100%)
	*cfb*	vagina	100%	(87.2–100%)	96.9%	(91.2–99.4%)
		rectum	90.9%	(70.8–98.9%)	99%	(94.7–100%)

**Table 3:** Carriage of GBS established by culture with preincubation in TH broth medium and NAAT-based methods (conventional PCR with the use of *atr* primers and real-time PCR with the use of *cfb* primers).

	Culture Methods	NAAT Directly form Clinical Materials			NAAT after Preincubation in TH Broth		
		*atr*	*cfb*	*atr + cfb*	*atr*	*cfb*	*atr + cfb*
vagina	7.14% (*n* = 6)	11.90% (*n* = 10)	8.33% (*n* = 7)	10.71% (*n* = 9)	7.14% (*n* = 6)	8.33% (*n* = 7)	7.14% (*n* = 6)
rectum	1.19% (*n* = 1)	4.76% (*n* = 4)	3.57% (*n* = 3)	4.76% (*n* = 4)	1.19% (*n* = 1)	0% (*n* = 0)	1.19% (*n* = 1)
vagina + rectum	14.29% (*n* = 12)	3.57% (*n* = 3)	3.57% (*n* = 3)	5.95% (*n* = 5)	15.48% (*n* = 13)	14.29% (*n* = 12)	16.67% (*n* = 14)
total GBS positive	22.62% (*n* = 19)	20.24% (*n* = 17)	15.48% (*n* = 13)	21.43% (*n* = 18)	23.81% (*n* = 20)	22.62% (*n* = 19)	25% (*n* = 21)
total GBS negative	77.38% (*n* = 65)	79.76% (*n* = 67)	84.52% (*n* = 71)	78.57% (*n* = 66)	76.19% (*n* = 64)	77.38% (*n* = 65)	75% (*n* = 63)

## Data Availability

The data presented in this study are available upon reasonable request to the corresponding author.
